# Toward Conductive Polymer-Based Soft Milli-Robots for Vacuum Applications

**DOI:** 10.3389/frobt.2019.00122

**Published:** 2019-11-28

**Authors:** Amine Benouhiba, Patrick Rougeot, Morvan Ouisse, Cédric Clévy, Nicolas Andreff, Kanty Rabenorosoa

**Affiliations:** FEMTO-ST Institute, Université Bourgogne Franche-Comté, National Center for Scientific Research, Besançon, France

**Keywords:** soft milli-robots, electroactive polymers, conducting polymers, micromanipulation, vacuum environment

## Abstract

For the last two decades, the development of conducting polymers (CP) as artificial muscles, by materials researchers and chemists, has made establishing a reliable and repeatable synthesis of such materials possible. CP-based milli-robots were mostly unknown in soft robotics, however, today, they play a vital role in robotics and smart materials forums. Indeed, this subclass of soft robots has reached a crucial moment in their history, a moment where they can display rather interesting features, based on established foundations in terms of modeling, control, sensing, and planning in various applications. The purpose of this paper is to present the potential of conductive polymer-based soft milli-robots as high-performance devices for vacuum applications. To that end, a trilayer polypyrrole-based actuator was first used inside a scanning electron microscope (SEM), characterized for different applied voltages, over a relatively long period. Additionally, the tip positioning of the cantilever was also controlled using a closed-loop control. Furthermore, as a proof of concept for more complex soft milli-robots, an S-shaped soft milli-robot was modeled, using a hybrid model comprised of two models; a multi-physics model and a kinematic model. It was then fabricated using laser machining and finally characterized using its tip displacement. polypyrrole-based soft milli-robots proved to have tremendous potential as high-performance soft robots at the microscale for a wide range of applications, including SEM micro-manipulation as well as biomedical applications.

## 1. Introduction

Nature has always inspired robotic designs and concepts. Animals, for instance, such as elephants (Trivedi et al., 2008) and octopuses (Laschi et al., 2012), can exhibit continuous large deformations with variable stiffness and an ability to manipulate different objects of various sizes and geometries. Likewise, plants can also grow and adapt to their soundings (Hawkes et al., 2017; Mazzolai, 2017). In the last two decades, the interest in soft robotics has significantly increased. Naturally, since traditional manipulator robots have rigid bodies with limited motion and degrees of freedom, they cannot display such behavior. Meanwhile, soft robots offer more dexterity with distributed deformation and versatility through shape-shifting. They can therefore easily navigate through constrained spaces and follow a tortuous path (Trivedi et al., 2008; Hawkes et al., 2017). They are mainly made of soft material, components, and monolithic active structures, which ensure safe and secure interaction with their immediate environment (Alici, 2018).

More to the point, recent technological advancements in numerous fields of applications, especially biomedical (De Greef et al., 2009; Rafii-Tari et al., 2014; Chikhaoui et al., 2016) and micro-manipulations (Zhang et al., 2019), have heavily relied on achievements in microtechnology and nano-science (Hu et al., 2018). As a result, the developments of new equipment and devices, such as micro-soft actuators (De Volder and Reynaerts, 2010; Paek et al., 2015) and micro-soft robots (Kim, 2017), to further reach this goal and improve small-scale manipulations, is of high interest to engineers and researchers. Furthermore, trying to scale down conventional robots to nano/micro-systems can be very problematic. For instance, it is very challenging to miniaturize the electromagnetic motor. Additionally, the existence of friction and backlash in prismatic/revolute joints can drastically decrease the performances of the nano/micro-robots (Goldfarb and Celanovic, 1999; Aw et al., 2013). However, soft robots that rely on smart materials (Moghadam et al., 2015; Chikhaoui et al., 2018; Cao et al., 2019; Minaminosono et al., 2019), do not have such limitations.

Commonly used materials for such purposes are Electro-Active Polymers (EAPs). They are a relatively new class of materials that can act as actuators (McGovern et al., 2009; Mutlu et al., 2013, 2014, 2015, 2016), as well as sensors (Bar-Cohen and Bar-Cohen, 2004; Vidal et al., 2004). Ionic Metal Polymer Composites (IPMCs) and Conductive polymers (CPs), which display an interesting performance in both actuation and sensing, are classified as EAPs (Smela, 2003; Moghadam et al., 2014; Bar-Cohen and Anderson, 2019). IPMCs and CPs offer several advantages, thanks to their (i) large strain, (ii) bio-compatibility, (iii) reduced space requirement (easily integratable), (iv) relatively low activation voltage, and (v) inherent compliance (Madden et al., 2004; Wu et al., 2006). Compared to the use of traditional active materials such as piezoelectric materials, which have limited displacement, a high activation voltage, and in some cases can even be toxic, CPs exhibit high potential in a wide range of applications.

In this paper, we propose to investigate the potential of Polypyrrole-based CPs robots for micro-manipulation, in a vacuum environment, and with complex forms. To do so, we started by testing the working behavior of a trilayer Polypyrrole-based cantilever inside a Scanning Electron Microscope (SEM). We characterized its performance and controlled its tip positioning in a closed-loop through visual feedback. Furthermore, in order to investigate the fabrication of more complex designs and configurations for micro-manipulation, we fabricated an S-shaped soft milli-robot. A CO_2_ laser machine and trilayer polypyrrole sheets were utilized. The S-shaped robot was also modeled using a hybrid model, introduced previously for CP based active origami (Benouhiba et al., 2018a), a multi-physics model for the deformation of the Polypyrrole-based CPs and a Kinematic model, in order to reconstruct the entire deformed shape. The model was experimentally validated using the systems tip displacement. The main objective of the following work is to assess the potential of Polypyrrole-based CPs soft milli-robots, to produce high-performance devices for micro-manipulation in a vacuum environment (Lewin, 1990).

The paper is divided into five sections. Section 2 describes the hybrid model of the S-shaped design, a combination between a multi-physics model and a Kinematic model. Section 3 details the fabrication process as well as the experimental setup utilized for this work. Section 4 discusses the performance of the trilayer polypyrrole actuator in a vacuum environment (inside SEM), for different applied voltages, as well as during closed-loop control tests. It also presents a proof of concept of complex designs (an S-shaped design) that can be used inside SEM applications. Finally, section 5 concludes the paper.

## 2. Model

The model used here to simulate the behavior of soft milli-robots is a hybrid model that combines two different models; (i) a multi-physics model and (ii) a kinematic model.

### 2.1. Multi-Physics Model

**Figure 1:**
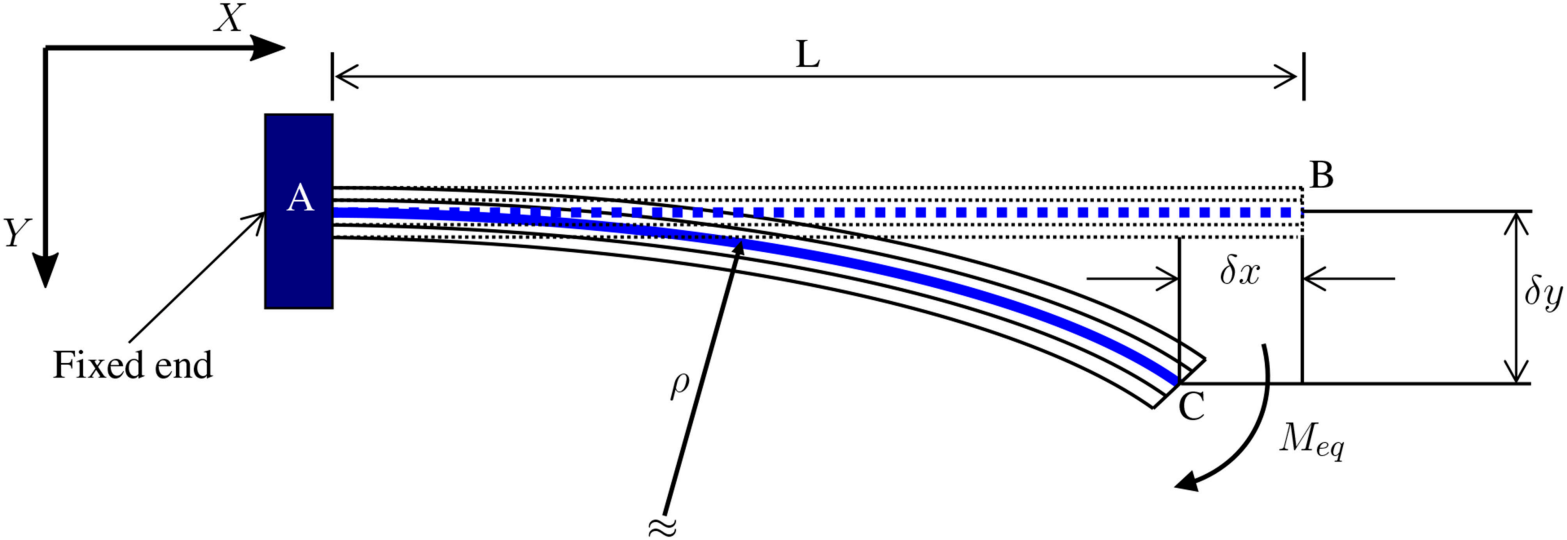
Description of large deflection of multilayer cantilever adapted from Kim (2008).

The multi-physics model is utilized to predict the behavior of active segments during large deformation (when an electric field is applied). Moreover, it is a large deflection model for multi-layer cantilevers (as shown in Figure 1); therefore, it is not applicable for passive segments, as they are not cantilever shaped. The model is derived from the work of Kim (2008). Several assumptions were made here:

– the material of each layer remains linearly elastic;
– two consecutive layers are perfectly bonded;
– the radius of the curvature ρ of the multimorph cantilever induced by the combined effect of all stresses is much greater than its thickness.

According to Kim (2008), the tip deflection of the multilayer cantilever is as follows:

(1) $$\begin{array}{l} \delta x=L-\rho .sin\;(\frac{L}{\rho }) \end{array}$$

(2) $$\begin{array}{l} \delta y=\rho \left(1-cos\;(\frac{L}{\rho })\right) \end{array}$$

Ultimately, during large deformation Equations (1, 2) it is assumed that the multilayer cantilever maintains its linear elastic properties. This assumption is indeed true for typical thin Micro-Electro-Mechanical Systems (MEMS), which is certainly the case here.

**Figure 2:**
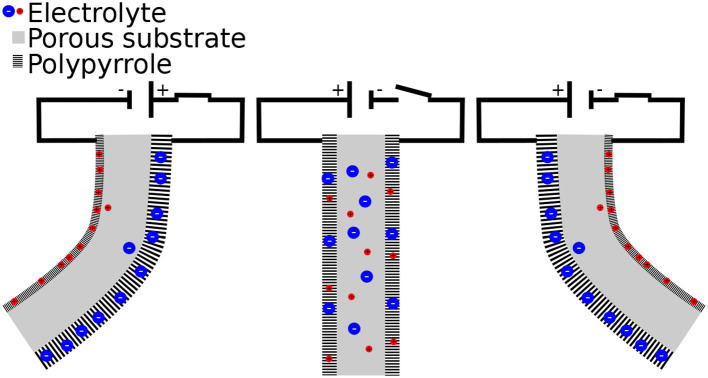
Working principle of trilayer polypyrrole micro-actuator.

Moreover, the system here is a polypyrrole-based trilayer electro-active micro-actuator, also known as active segments. Active segments contain three different layers: two polypyrrole active layers holding a PolyVinylidene DiFluoride (PVDF) porous passive layer between them. During the activation of this type of segment, the strain generated in the active layers, caused by the rushing of ions from one side to the other, induces an increase in the bending of the multilayer cantilever, thus transforming an electric field into a bending motion (as shown in Figure 2). The variable list is displayed in Table 1. Furthermore, the radius of the curvature ρ is derived according to Kim (2008) as such:

(3) $$\begin{array}{l} \rho =\frac{2RA^{-1}S}{2+RA^{-1}B} \end{array}$$

where:

(4) $$\begin{array}{l} R=\frac{-1}{E_{1}I_{1}+E_{2}I_{2}+E_{3}I_{3}}\left[\left(\frac{t_{1}}{2}\right)\left(t_{1}+\frac{t_{2}}{2}\right)\left(t_{1}+t_{2}\frac{t_{3}}{2}\right)\right] \end{array}$$

(5) $$\begin{array}{l} A=\left[\begin{array}{ccc} \frac{1}{E_{1}I_{1}} & \frac{-1}{E_{2}I_{2}} & 0\\ 0 & \frac{1}{E_{2}I_{2}} & \frac{-1}{E_{3}I_{3}}\\ 1 & 1 & 1 \end{array}\right],B=\left[\begin{array}{c} t_{1}+t_{2}\\ t_{2}+t_{3}\\ 0 \end{array}\right],S=\left[\begin{array}{c} \epsilon_{2}-\epsilon_{1}\\ \epsilon_{3}-\epsilon_{2}\\ 0 \end{array}\right] \end{array}$$

**Table 1:** List of model variables.

**Variables**	**Definition**	**Value**	**Unit**
*E*_1_/*E*_3_	Young's modulus of the first/third layers of PPy	80 × 10^6^	*Pa*
*E*_2_	Young's modulus of the second layer of PVDF	440 × 10^6^	*Pa*
*t*_1_/*t*_3_	Thicknesses of the first/third layers of PPy	7.5 × 10^−6^	*m*
*t*_2_	Thickness of the second layer of PVDF	55 × 10^−6^	*m*
*b*	Width of the multilayer cantilever	0.4 × 10^−3^	*m*
*L*	Length of the multilayer cantilever	3.1 × 10^−3^	*m*
α	Corrective factor	1.226 × 10^−1^	(*F*/*m*^2^)/(*C*/*m*^3^)
*C*	Volumetric capacitance	4 × 10^8^	*F*/*m*^3^
*DV*	Input voltage	0.1 < *DV* < 1	*v*

Since the second layer is passive, its strain ϵ_2_ in matrix S is null. Nevertheless, the strain of the two remaining active layers, first and third, is $\epsilon_{1}=2\alpha \frac{C.DV_{1}}{t_{1}bLE_{1}}$ and $\epsilon_{3}=2\alpha \frac{C.DV_{3}}{t_{3}bLE_{3}}$, respectively. Furthermore, as *DV*_1_ = −*DV*_3_, which is crucial to activate the trilayer micro-actuator, matrix S becomes:

(6) $$\begin{array}{l} S=\left[\begin{array}{c} 2\alpha \frac{C.DV}{t_{1}bLE_{1}}\\ 2\alpha \frac{C.DV}{t_{3}bLE_{3}}\\ 0 \end{array}\right] \end{array}$$

Additionally, by utilizing Equations (3–6), the radius of the curvature of the active segment, ρ can be calculated.

Lastly, once the radius of the curvature of the active segment ρ is determined, it is employed to calculate the tip deflection of the active segments, along the x- and y-axis, as pointed in Equations (1, 2). Nevertheless, for the sake of convenience, the fully developed equation of the tip deflection will not be detailed. More details about the model can be found in our previous work (Benouhiba et al., 2018b).

### 2.2. Kinematic Model

**Figure 3:**
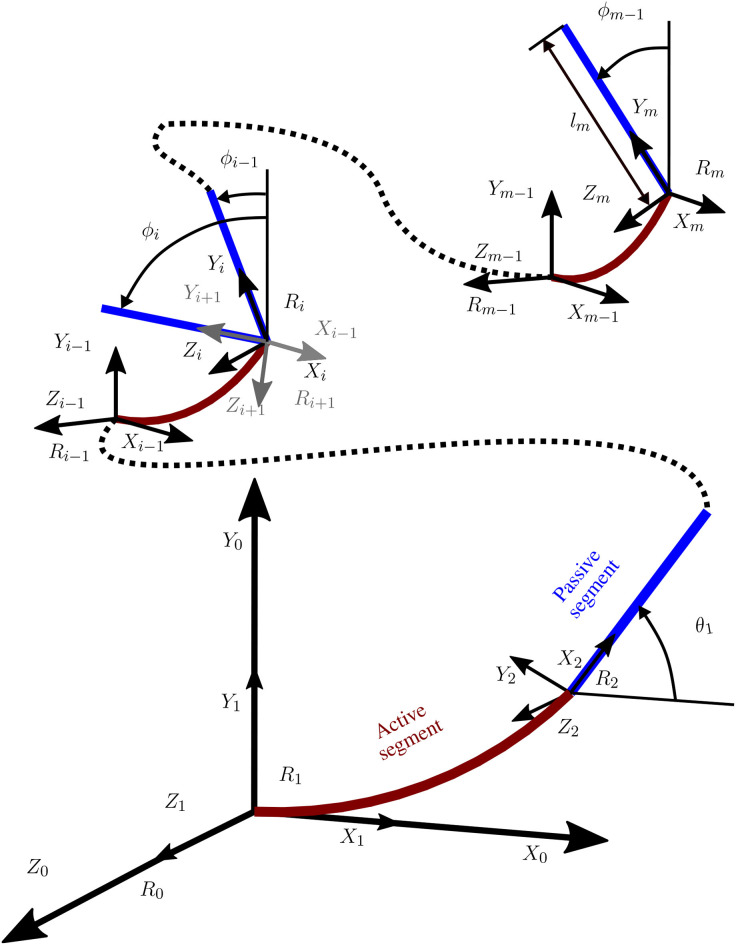
Coordinate system for the soft milli-robot.

For each applied voltage, the deformation of all active segments can be estimated using the multi-physics model. A kinematic model is added in order to reconstruct the complete form of the S-shaped system, using both active ($C_{A}^{i}$) and passive segments ($C_{P}^{i}$). The two types of segments can be represented in the global frame *R*_0_, as shown in Figure 3 using their frames by the following matrices, respectively:

(7) $$\begin{array}{l} C_{A}^{i}=\left[\begin{array}{cccc} 0 & x_{i,1} & \ldots & x_{i,n}\\ 0 & y_{i,1} & \ldots & y_{i,n}\\ 0 & z_{i,1} & \ldots & z_{i,n}\\ 1 & 1 & \ldots & 1 \end{array}\right], \end{array}$$

(8) $$\begin{array}{l} C_{P}^{i}=\left[\begin{array}{cc} 0 & x_{i}\\ 0 & y_{i}\\ 0 & z_{i}\\ 1 & 1 \end{array}\right], \end{array}$$

*x*_*i*_, *y*_*i*_, and *z*_*i*_ are the coordinates of the passive segment *i*, and *x*_*i, j*_, *y*_*i, j*_, and *z*_*i, j*_ are the coordinates of the point *j* from the curvature of the active segment *i*. The curvature of the active segments is discretized into *n* points, which means that *j* goes from 1 to *n*. The *n* coordinates for the curvature are actually obtained using the multi-physics model.

Once each matrix of the *m* segments of the model is determined, for a specific applied voltage, the Kinematic model is utilized to reconstruct the full shape of the soft milli-robot using appropriately defined homogeneous transformation matrices. In the case of a *i* − 1 active segment, the transformation matrix is:

(9) Mii−1=[cosϕicosθi −cosϕisinθisinϕixi−1,nsinθicosθi0yi−1,n−sinϕicosθi sinϕisinθicosϕizi−1,n0001],

Where θ_*i*_ is the rotation around the Z-axis and ϕ_*i*_ is the rotation around the Y-axis, of the segment *i*.

In the case of a *i* − 1 passive segment, the transformation matrix is:

(10) Mii−1=[cosϕicosθi −cosϕisinθisinϕixi−1sinθicosθi0yi−1−sinϕicosθi sinϕisinθicosϕizi−10001],

The representation of an *i* segment in the frame of an *i* − 1 segment is allowed by Equations (9, 10). However, in order to express the *i* segment in the global frame *R*_0_, the following matrix is needed:

(11) Mi0=∏j=1iMjj−1.

Finally, using Equations (8, 7, 11), the geometry of the soft milli-robot can be expressed in the global frame *R*_0_ using the concatenation of all the equations of the *m* different segments, as such:

(12)                                       SRm=[|1M0 CP,A1|  ...  |∏j=1iMjj−1 CP,Ai|  ...  |∏j=1mMjj−1 CP,Am|].

Since the full model goes beyond the scope of this paper it will not be discussed further here, however, more details about the two parts of the model, the multi-physics model, and the Kinematic model can be found in the work of Benouhiba et al. (2018a,b), respectively.

**Figure 4:**
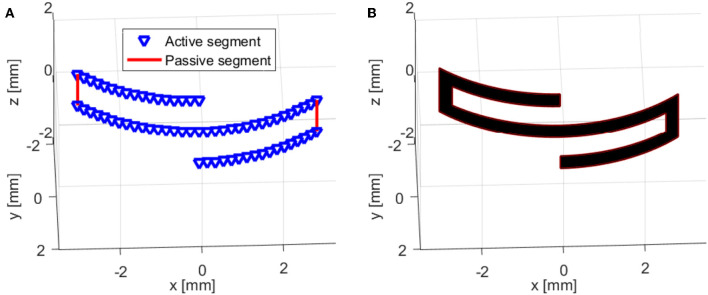
Representation of the hybrid model (multi-physics model coupled with a Kinematic model) for an S-shaped polypyrrole-based soft milli-robot: **(A)** the model contains four active segments and two passive segments, **(B)** another representation of the model (more realistic) where all the segments were replaced with rectangular shapes, which have a width of 0.4 mm (1:1 dimensions).

This model was used to simulate the behavior of a CP based S-shaped soft milli-robot. As mentioned before, The latter is considered to have two different types of segments; (i) active segments, which are cantilever-like segments, with a length of 3.1 mm and a width of 0.4 mm, and (ii) passive segments (as shown in Figure 4), which are small sheet-like segments with a size of 0.6 × 0.4 mm^2^. When a voltage is applied, active segments will bend, producing large deformations along its length, while passive segments will be too constrained, because of their sheet-like shape, to produce any noticeable deformation. Therefore, such segments are considered to be utterly passive, and their deformation is not considered by the model.

## 3. Fabrication and Experimental Setup

### 3.1. Fabrication

The fabrication process of the different Polypyrrole-based soft milli-systems is composed of two stages. First, conductive polymer (Polypyrrole-based) sheets are fabricated. The process starts with a double-sided coating (the cathodic sputtering process was utilized) of PVDF membrane Immobilion P from Milli-pore (product information: A 70% porosity with a pore size of 0.45 μ*m*). An anchor chromium layer is deposited first, followed by gold electrodes. Each side of the PVDF membrane contains 10 / 50 nm chromium to gold thickness ratio, respectively. Then, using a standard three-electrode electro-polymerization process, an additional polypyrrole layer was also deposited on both sides of the membranel. A 5 × 5 cm^2^ platinum wire mesh plate was used for the counter electrode, an Ag/AgCl electrode for the reference electrode, and the PVDF membrane for the working electrode. The polypyrrole layers were deposited at −20°, for 10 h and with a current density of 0.2 A/cm^2^. An OrigaFlex-OGF500 (from OrigaLys ElectroChem SAS), potentiostat was used, managed by OrigaMaster5^1^ software. The process is discussed in more detail in the work of Cot et al. (2016).

Once the Polypyrrole-based active sheets are fabricated, a CO_2_ laser cutter was utilized in order to fabricate the different configurations of the soft milli-systems. One of the major concerns of using the laser cutter was the potential damage caused by heat from the laser, thereby, drastically reducing the performance of the produced actuators. For that reason, a series of tests were carried out in order to determine acceptable parameters for the laser cutting process. In this work, an S-shaped configuration, as shown in **Figure 9a** was fabricated and tested.

### 3.2. Experimental Setup

For the characterization of the Polypyrrole-based electroactive soft milli-robot, a test bench was developed. It is managed by MATLAB Simulink software utilizing Visual Servoing Platform (ViSP) in addition to a dedicated block set cvlink^2^. A 3D printed part combined with machined and manually bent strips of thin stainless-steel sheets, were used to create a support for the milli-system, as well as, to provide electrical connections for the different electrodes, as shown in **Figure 9b**. A multi-function data acquisition module (USB-6211) from National Instruments was utilized in order to provide the needed electrical voltage. The module can manage voltages between ± 10 V with a high resolution (3.5 mV). As for the camera, an IEE 1394 Guppy Firewire, was used to capture the response of the soft milli-systems. The camera was placed across from the 3D printed supports, in a manner that visualizes the soft milli-robot from its thickness.

## 4. Results and Discussion

### 4.1. Inside SEM Manipulation

**Figure 5:**
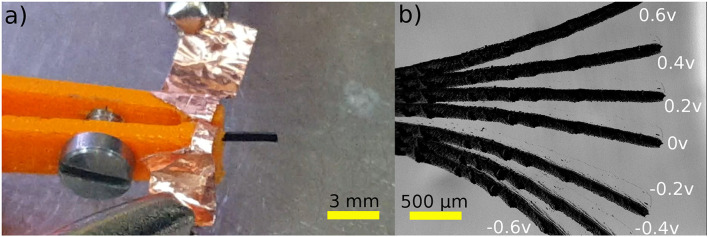
Polypyrrole-based micro-actuator for inside SEM procedures: **(a)** the experimental setup and, **(b)** the responses of the micro-actuator for different applied voltages, from –0.6 to +0.6 V with a step of 0.2 V.

As a proof of concept of a Polypyrrole-based milli-system designed for SEM manipulation, as well as other SEM procedures, a less complex configuration (cantilever) of trilayer Polypyrrole-based electroactive material (also known as conducting polymer) is proposed here. According to Wu et al. (2006), Polypyrrole-based micro-actuators can generate forces superior to 1 mN, which is more than enough force to manipulate micro/nano-objects inside a SEM. The 3 mm long cantilever, with a width of 400 μ*m* and a thickness of 106 μ*m* was inserted into a SEM (The used microscope is an Auriga 60 microscope produced by Zeiss with a big vacuum chamber that has a volume of 60 × 60 × 60 cm^3^, more details are available in Rauch et al., 2018), tested, characterized, and controlled using visual servoing. The cantilever was first mounted on a small 3D printed support, which contains copper tape to provide electrical connections, as shown in Figure 5a. Most of the support was then coated with a thin coat of gold (tens of nm) in order to prevent charge accumulation. Next, the support with the cantilever was inserted inside the SEM and was tested using different applied voltages, ranging from –0.6 V to +0.6 V with a step of 0.2 V. A superimposition of the different generated displacements, for the different applied voltages, and a magnification of 40 ×, is shown in Figure 5b. This result (very high displacement compared to the size of the cantilever and the values of the applied voltages) proves that the Polypyrrole-based milli-system has a similar performance inside The SEM than that of an open environment, if not better.

**Figure 6:**
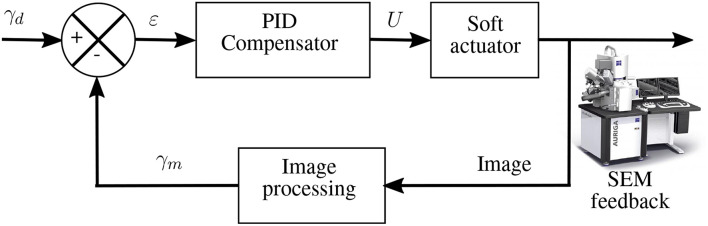
Block diagram for the visual servoing based angular control.

**Figure 7:**
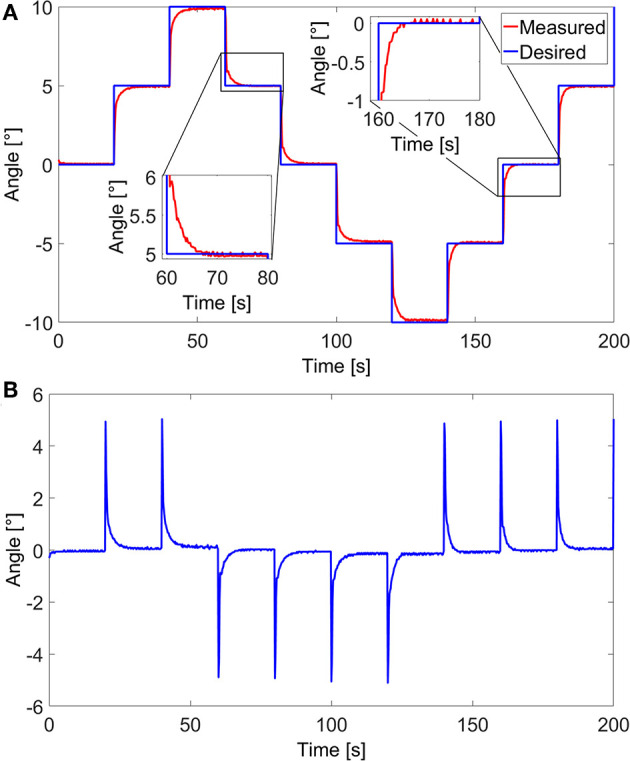
Closed loop control of the polypyrrole micro-actuator in the vacuum environment (inside SEM). **(A)** Angular response to a stairstep reference signal and **(B)** the corresponding error.

**Figure 8:**
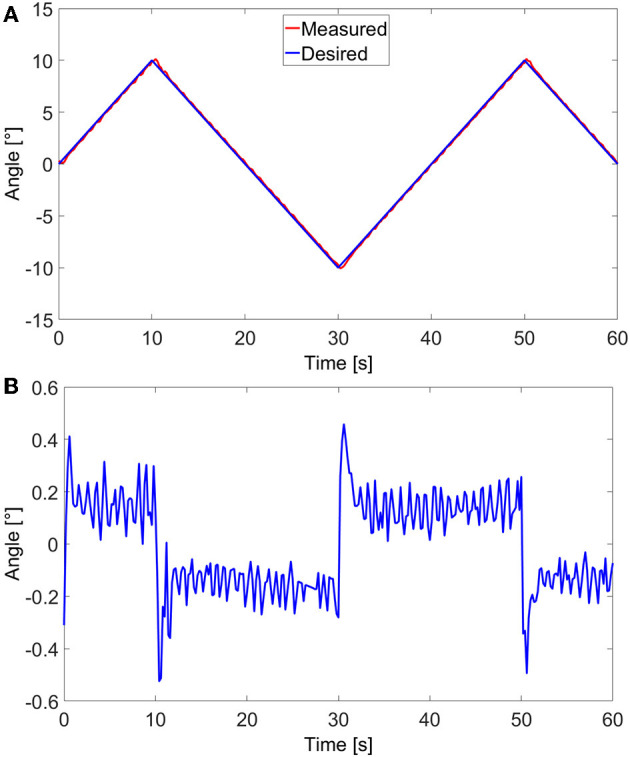
Closed-loop control of the polypyrrole micro-actuator in the vacuum environment (inside SEM): **(A)** angular response to a sawtooth reference signal and **(B)** the corresponding tracking error.

A closed-Loop control of the angular movement of the cantilever, inside the SEM, was also conducted. Visual tracking of the cantilever's tip was done using normalized cross-correlation as previously done using images from Optical Coherence Tomography (OCT) in Baran et al. (2017). The detected cantilever's tip is expressed in an angular position. The variation in the angular position γ is the variation between the fixed end/old tip position and fixed end/new tip position. Based on visual feedback, a closed-loop control scheme is proposed to control the end-effector's position accurately. The experimental tests were conducted using the control scheme in the block diagram presented in Figure 6. A PID controller was utilized to compensate for the estimated error between the desired angular position γ_*d*_ and the measured angular position γ_*m*_. The response of the soft milli-actuator in respect to the control reference signals, stairstep, and sawtooth signals, as well as their errors are displayed in Figures 7, 8, respectively.

The maximum angle for both signals was limited to ±10° in order for all parts of the micro-actuator, including its tip, to stay visible in the image. Furthermore, in the case of the stairstep control signal, the response time of the micro-actuator could have been faster if the applied voltages *U* were not limited to ±0.5 V; however, using any higher voltages may lead to an overshoot, which, in this case means going out of the frame and losing track of the system. That said, once the latter reaches the signal, it stays firmly on it, with a root-mean-square static error of 0.08°, which, compared to the maximum angular variation, is only an error of 0.4%. Consequently, we find these results to be satisfactory. Moreover, thanks to the high error values at the beginning of each step, the root-mean-square of the overall error of the micro-actuator in this case, has a value of 0.71°, which is high compared to only 0.17° for that of a sawtooth signal. Nonetheless, the error is still relatively small compared to the maximum angle variation (only 3.5%) and the overall size of the cantilever. The values of the standard, maximum, and minimum errors, for both stairstep and sawtooth signals are available in Tables 2, 3, respectively.

**Table 2:** Closed loop control error of the polypyrrole micro-actuator, for an angular response to a stairstep reference signal, in vacuum environment.

	**RMS (**°**)**	**STD (**°**)**	**MAX (**°**)**	**MIN (**°**)**
Test 1	0.71(3.5%)^*^	0.71(3.5%)^*^	5(25%)^*^	0(0.0%)^*^
Test 2	0.77(3.9%)^*^	0.77(3.9%)^*^	5(25%)^*^	0(0.0%)^*^
Test 3	0.75(3.8%)^*^	0.73(3.7%)^*^	5(25%)^*^	0(0.0%)^*^
Mean	0.74(3.7%)^*^	0.74(3.7%)^*^	5(25%)^*^	0(0.0%)^*^

* *Relative error compared to the maximum angle variation*.

**Table 3:** Closed loop control error of the polypyrrole micro-actuator, for an angular response to a sawtooth reference signal, in vacuum environment.

	**RMS (**°**)**	**STD (**°**)**	**MAX (**°**)**	**MIN (**°**)**
Test 1	0.17(0.9%)^*^	0.17(0.9%)^*^	0.5(2.5%)^*^	0(0.0%)^*^
Test 2	0.21(1.0%)^*^	0.21(1.0%)^*^	0.7(3.5%)^*^	0(0.0%)^*^
Test 3	0.16(0.8%)^*^	0.16(0.8%)^*^	0.8(4.0%)^*^	0(0.0%)^*^
Mean	0.18(0.9%)^*^	0.18(0.8%)^*^	0.6(3.3%)^*^	0(0.0%)^*^

* *Relative error compared to the maximum angle variation*.

### 4.2. Complex Shape

**Figure 9:**
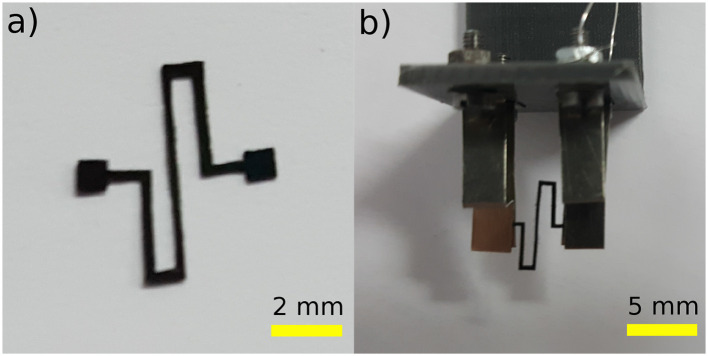
Prototype of a Polypyrrole-based milli-system, fabricated using laser machining: **(a)** an S-shaped system which was **(b)** mounted into an experimental setup, using 3D printed support and machined strips of thin stainless-steel sheets.

This subsection introduces a proof of concept of Polypyrrole-based soft milli-robots for open micro-manipulations. The milli-robot was fabricated through the laser machining (a CO_2_ laser machine was utilized) of trilayer Polypyrrole-based electroactive sheets. The S-shaped configuration has a line width of 0.4 mm (the width of the active material), and it occupies a surface of 6.2 × 2.4 mm^2^. It also has two opposite locations for electric activation; each one has a top and bottom electrode. Technically speaking, both top electrodes and bottom electrodes, for each location are connected; however, applying an electric field in both locations helps improve the response of the soft milli-robot. The latter was then mounted into its 3D printed support, and maintained in place by stainless steel pieces, as shown in Figure 9b. The stainless steel is also used to deliver the required electric field, without being affected by the system (reacting with the system), which may lead to negatively effecting its electric connections.

**Figure 10:**
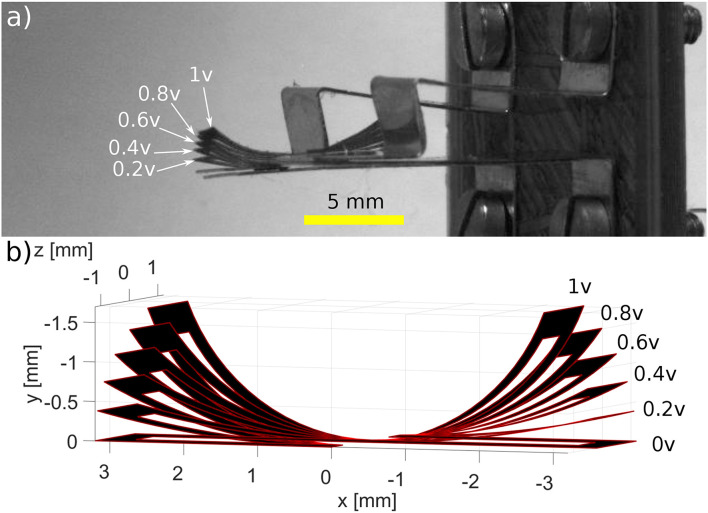
Comparison of **(a)** the experiment behavior of the S-shaped Polypyrrole-based milli-robot to **(b)** the behavior of the multi-physics model. Different voltages were used during the experiment, ranging from 0.2 to 1 V with a step of 0.2 V.

Once the system is appropriately mounted on the testing workbench, a series of experimental tests were conducted in order to characterize the performances of such systems. A voltage ranging from 0.2 to 1 V with a step of 0.2 V, was applied at both electrically connected locations. Each voltage was applied for a period of 25 s; then, a 30 s resting period was given to the system in order for it to regain its original form. The maximum deformation was then captured by the camera. A superimposition of the frames containing the maximum deformations can be shown in Figure 10. The maximum tip displacements of the robot were then calculated from the frames provided by the camera, using pixels to mm conversion. The experimental results were then compared to the results of the complex model (multi-physics and kinematic model).

**Figure 11:**
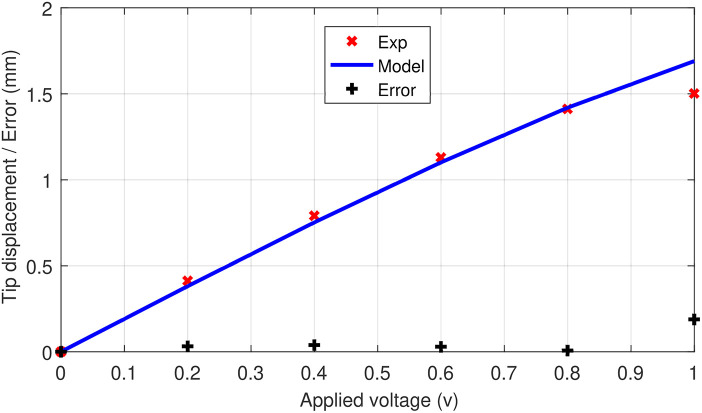
Comparison between experimental results and the proposed model, of tip displacement of the S-shaped soft milli-robot, for applied voltages ranging from 0 to 1 V.

A comparison between the behavior of the S-shaped soft system (the generated forms) and the model, for the different applied voltages, can also be seen in Figure 10. However, a more quantitative comparison, regarding the tip displacements of the milli-system, is shown in Figure 11. The S-shaped soft milli-robot was able to generate a maximum vertical tip displacement of 1.5 mm, which is compared to the size of the deformed part of the system (the 3.1 mm active segments) is a very high displacement ratio of 48%. The root mean square error between the experimental results and the model is of 88 μ*m*, which is 5.8% error compared to the maximum displacements of the tip. These values, as well as, the values of the standard, maximum and minimum errors are available in Table 4.

**Table 4:** Tip displacement's error of the S-shaped system model.

**RMS (μm)**	**STD (μm)**	**MAX (μm)**	**MIN (μm)**
88(5.8%)^*^	73(5.8%)^*^	187(12.4%)^*^	0.6(0.4%)^*^

* *Relative error compared to the maximum tip displacement*.

These results show that the model is able to predict, with high accuracy, the behavior of the soft milli-robot for the applied voltages, ranging from 0.2 to 0.8 V, however, once 0.8 V is reached the robot's deformation is saturated (an increase in voltage will only result in a minimal deformation). Therefore, the model is not able to accurately predict the deformation after this point.

## 5. Conclusion

In this paper, an investigation on the potential of Polypyrrole-based CPs systems in micro-manipulations, in open and controlled environments was conducted. The ability of functioning inside SEM of a CPs based milli-cantilever, with dimensions of 3.1 × 0.4 × 0.1 mm^3^, was tested and characterized for different applied voltages. Additionally, the angular motion of the beam bending milli-cantilever was controlled using a closed-loop control system. The tracking of the cantilever's tip was done using normalized cross-correlation. Then, as a proof of concept for a more complex system for micro-manipulations, an S-shaped system was modeled, fabricated, and characterized using its tip displacements. These results were also used in order to validate the model experimentally. The used model is a hybrid model containing two different models: a multi-physics model for simulating the bending motion of the active segments for an applied voltage, and a Kinematic model to reconstruct the full geometry of the S-shaped model afterward, using both active and passive segments.

One observation we made regarding internal SEM operations, is that the trilayer polypyrrole micro-actuator was able to function during the entirety of the 5 testing days, without a noticeable decline in its performance. It was surprising, because, usually after a short period (a few hours) their performance starts to decline (Cot et al., 2016). Therefore, it will be interesting to conduct a comparison between inside the SEM and open-air micro-actuators in the future works.

Numerous advantages of Polypyrrole-based soft milli-robots include its large deformation, low activation voltage, being easily integratable, and its inherent compliance. The results of this paper prove that they can be used in a vacuum environment (inside a SEM), be fabricated in complex shapes using laser machining, and can even be modeled using a hybrid model. Therefore, such active materials have the potential to be utilized for the fabrication of high-performance soft milli-robots for open environment and inside SEM micro/nano-manipulations, in addition to other applications, including biomedical applications.

## Data Availability Statement

All datasets generated for this study are included in the article/supplementary material.

## Author Contributions

All authors listed have made a substantial, direct and intellectual contribution to the work, and approved it for publication.

### Conflict of Interest

The authors declare that the research was conducted in the absence of any commercial or financial relationships that could be construed as a potential conflict of interest.

## Figures and Tables

**Table:** 

ρ	Radius of the curvature of the CP micro-actuator
*m*	Number of segments in the soft milli-robot
*i*	Index of the segment
*SR*	Matrix of the soft milli-robot
*C*_*P*_	Matrix used to express a passive segment
*l*	Length of a passive segment
*C*_*A*_	Matrix used to express an active segment
*n*	Number of points used to define the active segment
*j*	Index of the points of the CP actuator curvature
*x*_*n*_	Coordinate of the tip of the active segment along the X-axis
*y*_*n*_	Coordinate of the tip of the active segment along the Y-axis
*z*_*n*_	Coordinate of the tip of the active segment along the Z-axis
*M*	Homogeneous transformation matrix
θ	Rotation around Z-axis (folding angle)
ϕ	Rotation around Y-axis (the segment's orientation angle)

## Footnotes

^1^http://www.origalys.com/origasoft-logiciel-pc-origamaster-c2x19668303

^2^https://sourcesup.renater.fr/cvlink/
